# Provincial implementation supports for socio-demographic data collection during COVID-19 in Ontario’s public health system

**DOI:** 10.17269/s41997-021-00551-2

**Published:** 2021-08-09

**Authors:** Samiya Abdi, Caroline Bennett-AbuAyyash, Liane MacDonald, Karin Hohenadel, Karen O. Johnson, Pamela Leece

**Affiliations:** 1 Public Health Ontario, 480 University Avenue, Suite 300, Toronto, ON M5G 1V2 Canada; 2 Dalla Lana School of Public Health, University of Toronto, Toronto, Ontario M5T 3M7 Canada; 3 Department of Family and Community Medicine, University of Toronto, Toronto, Ontario M5G 1V7 Canada

**Keywords:** Health equity, Social determinants of health, Data collection methods, Professional education, Équité en santé, déterminants sociaux de la santé, méthodes de collecte de données, enseignement professionnel

## Abstract

**Setting:**

The Ontario government implemented a regulatory change to mandate the collection of socio-demographic (SD) data for individuals who tested positive for COVID-19. This change was informed by evidence of COVID-19’s disproportionate impact on marginalized communities and calls for broader collection of SD data. Given the scarcity of similar efforts, there is a significant knowledge gap around implementing standardized SD data collection in public health settings.

**Intervention:**

Public Health Ontario provided collaborative support for the implementation of SD data collection, grounded in health equity principles, evidence, and best practices. We supported the addition of SD fields in Ontario’s COVID-19 data collection systems, issued data entry guidance, hosted webinars for training and learning exchange, and published a resource to support the data collection process. The current focus is on building sustainability and quality improvement through continued engagement of public health units.

**Outcomes:**

By November 28, 2020, almost 80% of COVID-19 cases had information recorded for at least one SD question (individual questions, range 46.8–67.0%). We hosted three webinars for the field, and the data collection resource was viewed almost 650 times. Practitioners continue to express needs for support on applying equity principles to data analysis and interpretation, and community engagement on data collection and use.

**Implications:**

Sharing knowledge on responsive implementation supports in collaboration with the field and using current evidence and guidance will strengthen public health practice for SD data collection. Laying this groundwork will also improve the likelihood of success and sustainability of these equity-focused efforts.

## Résumé

**Lieu:**

Le gouvernement de l’Ontario a appliqué une modification réglementaire exigeant la collecte de données sociodémographiques (SD) sur les personnes testées positives pour la COVID-19. Cette modification découle des preuves de l’impact disproportionné de la COVID-19 dans les populations marginalisées et appelle à une collecte élargie des données SD. Comme les démarches similaires sont rares, il y a de grandes lacunes au sujet de la mise en œuvre de la collecte de données SD standardisées dans les milieux de la santé publique.

**Intervention:**

Santé publique Ontario a collaboré à la mise en œuvre d’une collecte de données SD ancrée dans les principes, les données probantes et les pratiques exemplaires de l’équité en santé. Nous avons appuyé l’ajout de champs SD dans les systèmes ontariens de collecte de données sur la COVID-19, fourni des orientations sur la saisie des données, organisé des webinaires de formation et d’échange de connaissances et publié un document à l’appui du processus de collecte de données. La priorité est actuellement accordée au renforcement de la durabilité et à l’amélioration de la qualité grâce à la participation continue des bureaux de santé publique.

**Résultats:**

Au 28 novembre 2020, pour près de 80 % des cas de COVID-19, il existait des informations sur au moins une question SD (questions individuelles, intervalle de 46,8 à 67,0 %). Nous avions organisé trois webinaires pour le personnel de terrain, et notre document sur la collecte de données avait été consulté près de 650 fois. Les praticiens continuent à nous demander de l’aide pour appliquer les principes d’équité à l’analyse et à l’interprétation des données et pour faire participer les communautés à la collecte et à l’utilisation des données.

**Conséquences:**

Le partage des connaissances sur les supports de mise en œuvre adaptés, en collaboration avec le terrain et en faisant appel aux connaissances et aux orientations actuelles, renforcera la collecte de données SD dans la pratique en santé publique. En posant ces bases, nous améliorons aussi les chances de réussite et la durabilité de ces démarches axées sur l’équité.

## Introduction

In early popular discourse, COVID-19 was framed as “the great equalizer” (e.g., Jones & Jones, 2020) and slogans such as “we’re in this together” were commonly shared. However, research had already shown that pandemics compound and exacerbate inequities (Mein 2020). As data and knowledge on the pandemic grew, findings from the United States, the United Kingdom, and others confirmed that COVID-19 disproportionately impacts racialized communities (Public Health Ontario, 2020a). In line with historical and current evidence of systemic racism broadly in Ontario (Bill 114, 2017) and within the Canadian health care system (Canadian Public Health Association, 2018), early findings in Ontario indicated:

- Areas with highest ethnic diversity had higher hospitalization rates (4× higher), higher intensive care unit (ICU) admission rates (4× higher), and higher death rates (2× higher) compared with those with the lowest ethnic diversity (Public Health Ontario, 2020b);
- Ontario neighbourhoods with the highest material deprivation had higher hospitalization (69% higher), ICU admission (2× higher), and death rates (52% higher) compared with those with lowest material deprivation (Public Health Ontario, 2020c).

Despite this evidence, gaps persist in the integration of equity within COVID-19 reporting standards and reopening plans across multiple jurisdictions. In Canada, reporting and planning have mainly focused on general case counts, deaths, hospitalizations, and metrics such as R-naught. The absence of equity-based metrics can be partly explained by the current narrow or poor socio-demographic (SD) collection infrastructures, particularly around race-based data (Canadian Public Health Association, 2020).

### Demographic data collection in Canada

There are several Canadian examples of standardized SD data collection in health settings prior to the pandemic. In British Columbia, the provincial Aboriginal Self-Identification (ASI) project started in 2007 in Interior Health; since then, a few reports have used the data in health strategies and planning (Interior Health, n.d.). Another project in Winnipeg, Manitoba focused on the collection of language and ethnicity data (Bowen, Botton & Roy, 2011). Two other initiatives, one in Saskatoon and another in Toronto, explored a wider range of data elements. A pilot from Saskatoon assessed the feasibility and impact of SD data collection (Williams-Roberts et al., 2017). In one example from Ontario, the Toronto Central Local Health Integration Network (LHIN) mandated data collection on eight SD elements across a network of community health centres and hospitals (Sinai Health System, 2017). Many other examples for Ontario were covered in a report by Bates et al. (2017). Common lessons from those experiences are as follows: patient/client openness to sharing SD information, importance of embedding data collection into workflows and infrastructures, and the critical role of data collector training in successful and sustainable data collection.

As of December 2020, provincial SD data collection for people who test positive for COVID-19 has been implemented in Alberta, Manitoba, and Ontario. In Alberta, race-based data are being collected but as of December 1, 2020, the province has not released data summaries after citing issues with completeness (Fletcher, 2020). Data collection in Manitoba focuses on Indigeneity and race/ethnicity (Manitoba Health, 2020) but the data have yet to be made public as of November 13, 2020 (Thorpe, 2020). In Ontario, provincial data collection started in late June 2020 but was preceded by SD data collection in four local public health units (PHUs): Middlesex-London Health Unit, Ottawa Public Health, Peel Public Health, and Toronto Public Health (Public Health Ontario, 2020d). Provincial-level data have not been reported as of the time of writing but a few health units have made it available at the regional level (e.g., Region of Peel, 2020). These data are captured in a provincial database and not accessible to clinicians or for use in care. Quebec had committed to race-based data collection in May 2020 but this has not yet been implemented despite community-level efforts to fill in that gap (Kestler-D’Amours, 2020). To support race-based data collection efforts, CIHI has proposed pan-Canadian standards for race and Indigeneity data collection in health sectors (Canadian Institute for Health Information, n.d.). Therefore, the pandemic has been a catalyst for a number of shifts in Canada’s approach to collecting SD data among patients and clients in the health system (see Table 1).

**Table 1:** Current demographic data collection for COVID-19 cases

Province	SD elements	Start date
Government of Alberta	☑ Race	N/A
Government of Manitoba	☑ Indigeneity ☑ Race	May 2020
Government of Ontario	☑ Race ☑ Household income ☑ Household size ☑ First language(s) learned in childhood ☑ Official language(s)	June 2020

The Public Health Agency of Canada asserts that equity and supportive data structures are vital for ensuring health security during and beyond COVID-19 (Public Health Agency of Canada, 2020). Community organizers, evidence, and experience with health equity and COVID-19 tell us that building accountable and responsive plans necessitates the collection and use of robust and reliable data on race, Indigeneity, income, and additional SD variables. The work must also be done through strong collaboration with and leadership by impacted communities to ensure the safe and meaningful use of SD data.

## Setting

Ontario’s public health system involves close collaboration between provincial partners and its 34 local PHUs. The Ministry of Health (MOH) provides strategic leadership for the health system and is responsible for health legislation and regulations. Within the MOH, the Office of the Chief Medical Officer of Health develops public health strategies and policies, and sets the standards for local Boards of Health and PHUs. This includes standards with respect to health equity, and communicable disease prevention and control programs and surveillance. Public Health Ontario (PHO) provides scientific and technical information, advice, and support to the MOH and PHUs to inform decisions and action, with an explicit aim to help reduce health inequities (Public Health Ontario, 2019). PHO’s role includes conducting surveillance at the provincial level.

Throughout the pandemic response in Ontario, local PHUs, PHO and the MOH have focused on collecting, entering, analyzing, and disseminating public health surveillance data on COVID-19 (Public Health Ontario, 2020e). During the pandemic, these data have been captured in Ontario’s integrated Public Health Information System (iPHIS), local data systems, or the newer Case and Contact Management System (CCM). However, gaps in the data limited the ability to describe the socio-economic burden of COVID-19 in Ontario.

Calls for SD data collection were growing in Ontario prior to COVID-19. As the provincial government developed COVID-19 responses and evidence on the growing detrimental impact of COVID-19 on racialized communities in Canada emerged, impacted communities and community-based organizations strengthened their calls for standardized SD data (Labby, 2020). This was particularly the case for Ontario Black health care leaders (e.g., Robertson et al., 2020).

In the absence of individual-level SD data, PHO analyzed neighbourhood-level data in Ontario to examine the link between COVID-19-related outcomes and neighbourhood characteristics (see Public Health Ontario, 2020a, b). However, neighbourhood-level data do not reflect heterogeneity within neighbourhoods and have limitations for various analyses for monitoring trends and tracking changes. Without individual-level data, system-level action on equity would be challenging.

On June 25, 2020, the Ontario government made regulatory changes to mandate SD data collection on COVID-19 cases (Public Health Ontario, 2020f). On June 27, a notice was issued to PHUs in Ontario that the new SD data fields were available in iPHIS (and later CCM).

## Intervention

Our team undertook a range of collaborative efforts and diverse knowledge translation activities to support the implementation of COVID-19 SD data collection in Ontario. This work aligns with PHO’s mission to enable informed action to reduce health inequities and mandate to provide scientific and technical advice (Public Health Ontario, 2019). Our objectives included but were not limited to:

- Review and synthesize information on existing and applicable SD data collection efforts;
- Support the addition of the SD fields in Ontario’s COVID-19 data collection systems and issue data entry guidance;
- Provide education and support to PHUs on collecting and using SD data.

**Fig. 1:**
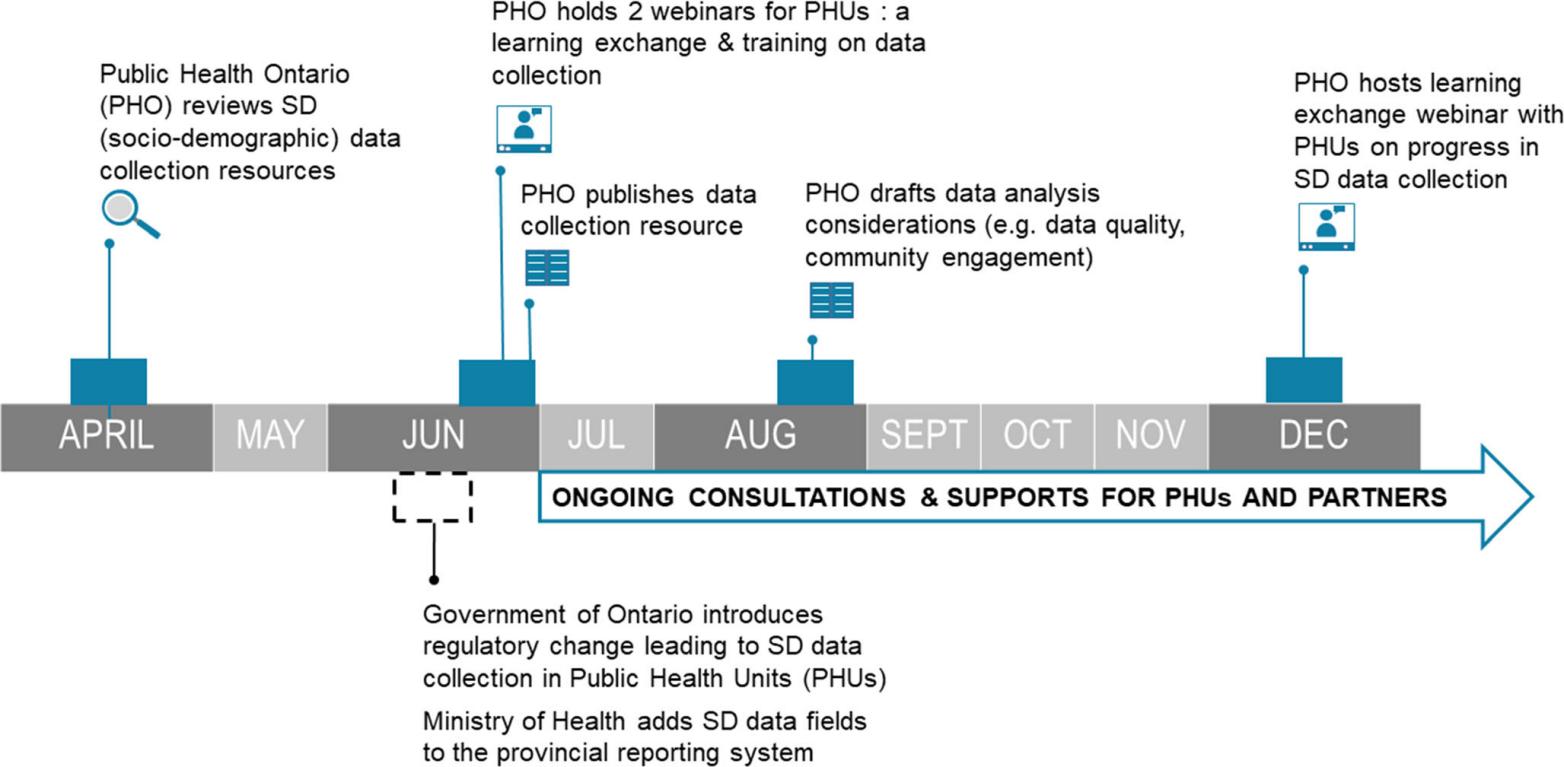
Timeline of key PHO activities and deliverables

The timeline of support activities and deliverables is outlined in Fig. 1. While undertaking these efforts, we recognize our role as supporters and facilitators of knowledge and education rather than as “experts” in the experiences and priorities of marginalized communities. Since the introduction of COVID-19 SD data collection in Ontario, there have been conversations on the risks of perpetuating harm and inequities using the data, as well as concerns about excluding community stakeholders and advocates (Walcott, 2020). Noting our primary role as provider of scientific and technical support to public health units, Ministry of Health, and other health system partners, local PHUs have been better positioned to leverage established local relationships that enable direct engagement with impacted communities. However, we engaged with racialized public health, data, and research experts, and stayed attuned to community calls to action around equitable and safe data collection. We continue to have internal conversations to reflect on how we can listen and support in ways that are appropriate, safe, and needed.

### Foundational supports

In April and May 2020, we reviewed current practices and considerations for the collection of SD data in Canada. This included the SD data collection jurisdictional scan covered in the introduction of this paper. Additionally, we were informed by existing data collection efforts in Ontario such as questions from Ontario’s Data Standards for the Identification and Monitoring of Systemic Racism (Ministry of the Solicitor General, 2020) and Toronto District School Board (Academic, Research, and Information Services, 2014). The review also spanned questions in development or in use at PHUs in Ontario, including Toronto Public Health, Peel Public Health, Ottawa Public Health, and Middlesex-London Health Unit. We also looked at community-based resources and consulted with community data experts on best practices and key considerations. Based on this review, we provided technical advice to the Ontario government on socio-demographic questions, including race and income.

Ontario subsequently finalized the COVID-19 SD questions and PHO continued to provide support by reviewing and testing the new data collection fields. Table 2 lists the SD questions that were added for individuals who test positive for COVID-19. The finalized race question was largely consistent with the CIHI interim standard to collect race-based data but did not include a “do not know” category. Additionally, an Indigenous category was not included at this time as there is ongoing work between the Ministry and Indigenous partners to discuss this further.

**Table 2:** Socio-demographic data collection questions

Category	Question	Response options (multiple responses allowed)
Race	Which race category best describes you?	Black East/Southeast Asian Latino Middle Eastern South Asian White Another race category Do not know Prefer not to answer
Household income	What was your total household income before taxes in 2019?	0–$29,999 $30,000–$49,999 $50,000–$69,999 $70,000–$99,999 $100,000–$149,999 $150,000 or more Do not know Prefer not to answer
Household size	Including yourself, how many family members live in your household?	________(people) Do not know Prefer not to answer
First language	What is the language that you first learned at home in childhood and still understand?	Drop-down list of languages Do not know Prefer not to answer
Official language(s)	In which of Canada’s official languages, English or French, are you most comfortable?	English French Both English and French Neither Do not know Prefer not to answer

### Capacity-building supports

As PHUs were about to start COVID-19 SD data collection province-wide, we supported capacity building through knowledge exchange and training. These supports (see Fig. 1) included learning exchange events, training, and resources. Our goal was to share evidence and best practices, integrate community and equity perspectives, and engage PHUs and their staff around SD data collection and use. Public-facing communications primarily happened through the government press release and news coverage. Our work on communicating with the patient/client focused on building staff capacity to have data collection conversations as part of case management phone calls with individuals who tested positive for COVID-19.

During the training, we emphasized that data collection should be grounded in an understanding of the historical context and the current realities facing Indigenous, Black, and racialized communities. Public accountability and community involvement are core components of responsible data collection and use. The purpose, uses, and disclosure of the collected information must be clearly communicated to impacted communities. Data collection should be linked to measuring and addressing health inequities. PHUs should also anticipate, manage, and mitigate potential unintended negative impacts on affected communities. More specifically, PHUs should ensure that data are stored, analyzed, and used in ways that do not cause harm or stigmatize.

PHO organized a data collection training webinar (June 2020) to support the introduction of data collection and build skills around data collection practices. The session covered core equity principles driving this work, including historical and current inequities. Attendees also learned about the SD questions (what they mean, why we collect), scripts, and data entry guidelines.

We also held two online learning exchanges that brought together PHUs to discuss emerging experiences, opportunities, and concerns. Following short PHU presentations, attendees participated in an interactive discussion with each other and the panelists. The first learning exchange (June 2020) focused on preparing and familiarizing PHUs with the process of implementing SD data collection. We invited three PHUs that started local SD data collection ahead of the provincial roll-out. Presenters shared their strategies for successful data collection, including training, workflows, engagement, and sustainability planning. The second learning exchange in December addressed the progress on data collection since early implementation. Reflecting a shift in focus from collection to analysis, three health units shared their experiences with improving data quality (e.g., missing data, data completeness), integrating an equity lens in this work, and initial efforts in analyzing the data to identify inequities in their regions. In the case of both events, PHO facilitated the discussion and collated questions for future use in training and support. Upon evaluation, discussion components were rated as one of the strongest elements of the events. In addition to events, the PHO team has prepared resources, including:

- Data collection resource document to support case managers in navigating the new SD data fields. The resource included a sample script that may be used during data collection, tips for case managers, frequently asked questions, and background information. This document was intended for use in conjunction with PHO’s technical data entry guide.
- Data analysis advice, organized into three parts: (1) data quality, (2) data summaries, (3) description of COVID-19 outcomes (e.g., death, hospitalization). This included the application of an intersectional lens in the analysis. For specific guidance on interpretation and messaging, we defer to the work of existing groups (e.g., the Black Health Equity Group blackhealthequity.ca), recognizing that this area of work should centre the perspectives of impacted communities rather than organizations holding the data. This approach would also help mitigate the harm and stigma that can result from sharing data about disproportionately impacted communities.

## Outcomes

### Data collection

**Fig. 2:**
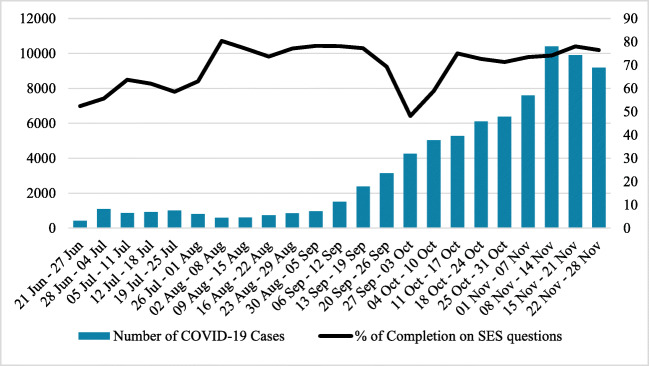
Percent of COVID-19 cases with at least one socio-demographic question completed and number of confirmed COVID-19 cases, by week, June 21–November 28, 2020

In the first full week of data collection, more than 50% of cases had information recorded for at least one of the SD questions (see Fig. 2). Following a decrease in early fall, by the week of November 22 to 28, almost 80% of cases had information recorded for at least one of the five new SD questions.

Table 3 shows completion rates for SD questions for the period of June 26–November 27. The data do not currently allow us to describe the reason for variation in completion rates, e.g., if the staff did not ask. There were no meaningful differences in completion rates by age or gender; however, long-term care residents were more likely to have missing information than individuals outside of long-term care (not presented), likely because information for residents is often provided by staff or proxies.

**Table 3:** Completion rate for socio-demographic questions, June 26–November 27

Question	Percent response
Race	67.0%
Household size	65.8%
Official language(s)	48.3%
Household income	47.2%
First language learned in childhood	46.8%

### Webinars and web metrics

The target audience for our webinars have been Ontario PHUs (34 in total). Within that group, we have seen representation from diverse professional roles (e.g., epidemiology/data analysis, management, nursing, health equity, health promotion, and other roles), as well as geographies (see Table 4).

**Table 4:** Webinar and web metrics summaries

Metrics	
Learning exchange webinar (June 24):	
Regional representation	30 of 34 PHUs
Number registered	177
Number of attendees	100
Number of times slides/recording was viewed*	197
Training webinar (June 30):	
Regional representation	32 of 34 PHUs
Number registered	224
Number of attendees	204
Number of times slides/recording was viewed*	269
Learning exchange webinar (December 15):	
Regional representation	18 of 34 PHUs in Ontario, 1 health agency in Quebec, 1 health agency in BC
Number registered	78
Number of attendees	50
Number of times slides/recording was viewed*	N/A
Number of times the data collection resource was viewed*	649

*To November 30, 2020

### Learning and support needs

Webinars have been effective tools to gauge future needs, identify knowledge gaps, and inform training and resources development. Education needs included training on data collection methods and workflows, community engagement in data collection and use, and responding to racism and harmful language during data collection. Other needs were guidance on applying equity to data analysis and interpretation, as well as direction on Indigenous data collection. Requests for support from PHUs were primarily related to data entry and data quality checks rather than operational pieces around implementation.

## Implications

PHO supported implementation of SD data collection and use, in the context of a recognized need, steep learning curve for this practice in public health in Ontario, and a constantly shifting pandemic. The immediate implications of this work include strengthening public health practices in SD data collection and use, driven by evidence and community perspectives. There are also implications for strengthening the sustainability of these equity-focused efforts. This work in Ontario, through wide partnerships within the health system, provides a blueprint for other jurisdictions or provinces considering implementation and support for SD data collection. Its potential to encourage others is evident in the interest expressed to PHO from other provinces and groups.

We acknowledge that rapid timelines added restrictions and limitations to our work. We had limited opportunities for outreach and engagement with PHUs to understand needs prior to SD data collection. Similar limitations applied to outreach and collaboration with other data collection stakeholders on how to align our data collection resources with others in the field. For example, our resource document included minimal information on the social aspect of collecting these data, including potential discomfort of staff or clients. Other knowledge exchange methods could potentially provide more appropriate teaching or skill development, such as videos modelling conversations. The support for implementation was not designed using a formal implementation framework or in a systematic way to address barriers and facilitators to implementation of SD data collection. Further, we have not conducted formal evaluation on the processes or outcomes involved in providing implementation support.

At the time of this paper, a local example of action is Toronto Public Health, which had started community-based consultations to inform plans for enhanced testing, pop-up centres, and tailored health promotion messages (Toronto Public Health, 2020). Lessons can also be gleaned from jurisdictions such as California, where data were used to develop an equity metric and set performance goals around reducing COVID-19 inequities (Public Health Ontario, 2020g).

As data collection and use evolve, our work will further expand into supporting practice in addressing health equity through data use. This includes using data to inform public health efforts in areas such as outbreak management and community support. At the organizational level, this work can support the use of SD data in program planning and implementation. At the system level, it can include data use in resource redistribution, tailored action, and policy change.

## Conclusion

Public Health Ontario provided a range of support for the implementation of socio-demographic data collection across Ontario’s public health units, a period which also overlapped with the unprecedented pressures of a pandemic. We provided technical support (e.g., for adding SD fields in Ontario’s COVID-19 data collection systems), learning exchanges between PHUs, training for data collectors, and other resources. During this period, we continued to engage practitioners across the province and in various roles in PHUs, community health, data expertise, and others. We continue to explore key support needs and engage PHUs to build sustainable, equitable, and impactful data collection systems to inform program and policy decisions to address health equity.

### Implications for policy and practice

What are the innovations in this policy or program?

- We provided scientific advice and knowledge translation activities to support the introduction of COVID-19 socio-demographic (SD) data collection by adapting resources from other Canadian health settings.
- This work contributes to filling gaps in SD collection infrastructures in the provincial and national health systems, particularly around race-based data.
- The collection and use of robust and reliable data on race, household size, income, and additional SD variables sets the foundation for building accountable and responsive policy and programs.

What are the burning research questions for this innovation?

- There is a need for research and evaluation on tools and methods used in supporting SD data collection in public health nationally and internationally, applying an implementation framework.
- Further study is needed on implementation supports and knowledge exchange methods for teaching and skill development in SD data collection and use, driven by equity-centred evidence and community perspectives.
- There is also a need to understand interventions to support practice in addressing health equity through data use, including its use to inform public health efforts, SD data in program planning and implementation, and resource redistribution, tailored programs, and policy change.

## Data Availability

Not available.

## Code availability

N/A
